# Genomic and Seasonal Variations among Aquatic Phages Infecting the Baltic Sea Gammaproteobacterium *Rheinheimera* sp. Strain BAL341

**DOI:** 10.1128/AEM.01003-19

**Published:** 2019-08-29

**Authors:** E. Nilsson, K. Li, J. Fridlund, S. Šulčius, C. Bunse, C. M. G. Karlsson, M. Lindh, D. Lundin, J. Pinhassi, K. Holmfeldt

**Affiliations:** a Faculty of Health and Life Sciences, Department of Biology and Environmental Science, Centre for Ecology and Evolution in Microbial Model Systems, Linnaeus University, Kalmar, Sweden; University of Illinois at Chicago

**Keywords:** Baltic Sea, bacteriophage, genomics, temporal variation

## Abstract

Knowledge in aquatic virology has been greatly improved by culture-independent methods, yet there is still a critical need for isolating novel phages to identify the large proportion of “unknowns” that dominate metagenomes and for detailed analyses of phage-host interactions. Here, 54 phages infecting *Rheinheimera* sp. strain BAL341 (*Gammaproteobacteria*) were isolated from Baltic Sea seawater and characterized through genome content analysis and comparative genomics. The phages showed a myovirus-like morphology and belonged to a novel genus, for which we propose the name *Barbavirus*. All phages had similar genome sizes and numbers of genes (80 to 84 kb; 134 to 145 genes), and based on average nucleotide identity and genome BLAST distance phylogeny, the phages were divided into five species. The phages possessed several genes involved in metabolic processes and host signaling, such as genes encoding ribonucleotide reductase and thymidylate synthase, *phoH*, and *mazG*. One species had additional metabolic genes involved in pyridine nucleotide salvage, possibly providing a fitness advantage by further increasing the phages’ replication efficiency. Recruitment of viral metagenomic reads (25 Baltic Sea viral metagenomes from 2012 to 2015) to the phage genomes showed pronounced seasonal variations, with increased relative abundances of barba phages in August and September synchronized with peaks in host abundances, as shown by 16S rRNA gene amplicon sequencing. Overall, this study provides detailed information regarding genetic diversity, phage-host interactions, and temporal dynamics of an ecologically important aquatic phage-host system.

**IMPORTANCE** Phages are important in aquatic ecosystems as they influence their microbial hosts through lysis, gene transfer, transcriptional regulation, and expression of phage metabolic genes. Still, there is limited knowledge of how phages interact with their hosts, especially at fine scales. Here, a *Rheinheimera* phage-host system constituting highly similar phages infecting one host strain is presented. This relatively limited diversity has previously been seen only when smaller numbers of phages have been isolated and points toward ecological constraints affecting the *Rheinheimera* phage diversity. The variation of metabolic genes among the species points toward various fitness advantages, opening up possibilities for future hypothesis testing. Phage-host dynamics monitored over several years point toward recurring “kill-the-winner” oscillations and an ecological niche fulfilled by this system in the Baltic Sea. Identifying and quantifying ecological dynamics of such phage-host model systems *in situ* allow us to understand and study the influence of phages on aquatic ecosystems.

## Abstract (precis)

Phages are important in aquatic ecosystems as they influence their microbial hosts through lysis, gene transfer, transcriptional regulation, and expression of phage metabolic genes. Still, there is limited knowledge of how phages interact with their hosts, especially at fine scales. Here, a *Rheinheimera* phage-host system constituting highly similar phages infecting one host strain is presented. This relatively limited diversity has previously been seen only when smaller numbers of phages have been isolated and points toward ecological constraints affecting the *Rheinheimera* phage diversity. The variation of metabolic genes among the species points toward various fitness advantages, opening up possibilities for future hypothesis testing. Phage-host dynamics monitored over several years point toward recurring “kill-the-winner” oscillations and an ecological niche fulfilled by this system in the Baltic Sea. Identifying and quantifying ecological dynamics of such phage-host model systems *in situ* allow us to understand and study the influence of phages on aquatic ecosystems.

## INTRODUCTION

Viruses are the most abundant biological entities on the planet (1). With an approximate abundance of 10^7^ viral particles ml^−1^ in surface waters of the global oceans, they outnumber their microbial hosts approximately 10-fold (2). Through infection and lysis of 20% of the bacterial community on a daily basis, viruses that infect bacteria (bacteriophages, or phages for short) are important factors influencing bacterial mortality and genetic diversity (3). Lately, high-throughput sequencing techniques have improved our knowledge regarding the viral communities, including viruses that infect so-far uncultured hosts. Metagenomic analyses have unraveled large-scale spatial variations within viral populations (4, 5), shed light on virus-host dynamics (6, 7), and predicted the functional gene potential of viral communities (8, 9). Moreover, single-cell genomics (10) of phage-host pairs from natural communities and viral tagging (11), where host-specific metagenomes are analyzed, have uncovered viral diversity and host interactions on finer scales. Since the discovery of auxiliary metabolic genes (AMGs) (12–14), which typically are metabolic genes transferred from the host’s genome to the phage genome that enable continued or increased phage replication during infection (15), the functional capacity of viral communities and the potential of these genes to influence biogeochemical cycles have gained increased interest.

The isolation of phage-host pairs is essential to elucidate fine-scale patterns of phage-host interactions. For example, different phages infecting the same bacterial species can display a wide range of infection abilities, both on a qualitative (whom they infect) and quantitative (how well they infect) scale (16). In addition, microdiversity within viral species has the ability to affect not only host range but also replication efficiency, seen, e.g., as burst size (17). Further, a viral strain can have different replication efficiencies on different host strains (17, 18) due to differences in the host transcriptional (19) and translational (20) responses. Thus, the knowledge gained from phage isolation and experimental studies is imperative to fully understand phage-host systems and hypotheses that derive from sequencing methods such as metagenomics, particularly with regard to viral diversity and ecological importance. This is especially important for nonmodel systems since we do not know how they differ from model systems before they have been isolated.

Clinically important phages infecting different gammaproteobacterial species, including, e.g., *Escherichia* spp. and *Pseudomonas* spp., are among the most well-studied phage-host systems in the world (21). However, in marine environments, phages infecting *Gammaproteobacteria* have received less attention, and research has been focused mainly on phages infecting *Pseudoalteromonas* (22–24) or *Vibrio* (13, 25, 26). These isolated *Pseudoalteromonas* (27, 28) and *Vibrio* (26) phages display a large diversity, with phages belonging to a variety of genera and families, which is similar to what has been seen for *Escherichia* (29) and *Pseudomonas* (30) phages. In addition, a podovirus and a myovirus, respectively, have been identified infecting the uncultured gammaproteobacterial SAR92 and SAR86 clades using single amplified genomics (10). However, information regarding phages infecting other *Gammaproteobacteria* species is sparse.

Members of the bacterial genus *Rheinheimera* exist in various environments, and they have been found in soils (31) and aquatic habitats, including both freshwater (32) and marine (33) environments. They have also been shown to proliferate in more extreme environments, for example, in iron backwash sludge (34), and are able to degrade hydrocarbons such as *n*-alkanes (35). Bacteria within this genus have been detected in the brackish Baltic Sea (36), and transplant experiments with shifting salinities have noted stabilizing or increasing abundances as adjustment effects (37). Even though species in the *Rheinheimera* genus are widespread, to our knowledge, no phages infecting these species have been isolated.

The aim of this study was to isolate and characterize aquatic phages infecting environmentally important bacteria and to increase our knowledge about phage diversity and the ecological relevance of phages within aquatic ecosystems. We present 54 genetically similar phages that are distinct from previously described phages and infect the Baltic Sea isolate *Rheinheimera* sp. strain BAL341. We propose that these phages are assigned to a novel viral genus, *Barbavirus*, consisting of five species which contain genes potentially involved in various metabolic pathways. The phages’ prevalence is evident by metagenomic recruitment, and their temporal abundances coincide with the host’s abundance in late summer.

## RESULTS

### Genomic characteristics.

**FIG 1:**
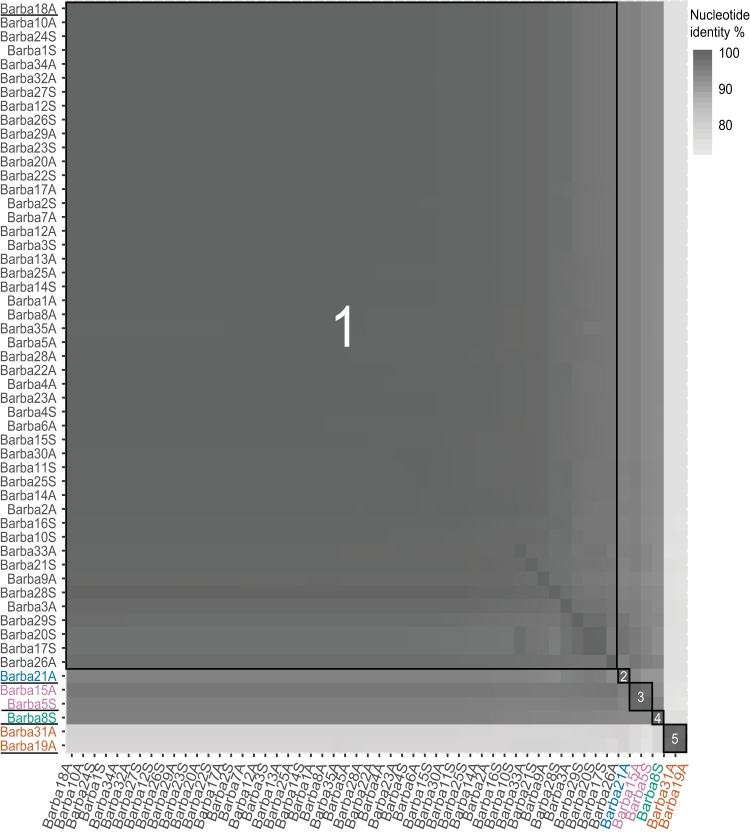
Pairwise blastn comparisons of average nucleotide identities across entire genomes were calculated using gegenees (version 3.0.0) (settings: fragment size of 500 and step size of 500) (103). The isolates belonging to the different species are boxed, and the names are color coded as follows: black, *Rheinheimera* virus Barba18A; blue, *Rheinheimera* virus Barba21A; pink, *Rheinheimera* virus Barba5S; green, *Rheinheimera* virus Barba8S; orange, *Rheinheimera* virus Barba19A. The type phages are underlined on the left axis.

**FIG 2:**
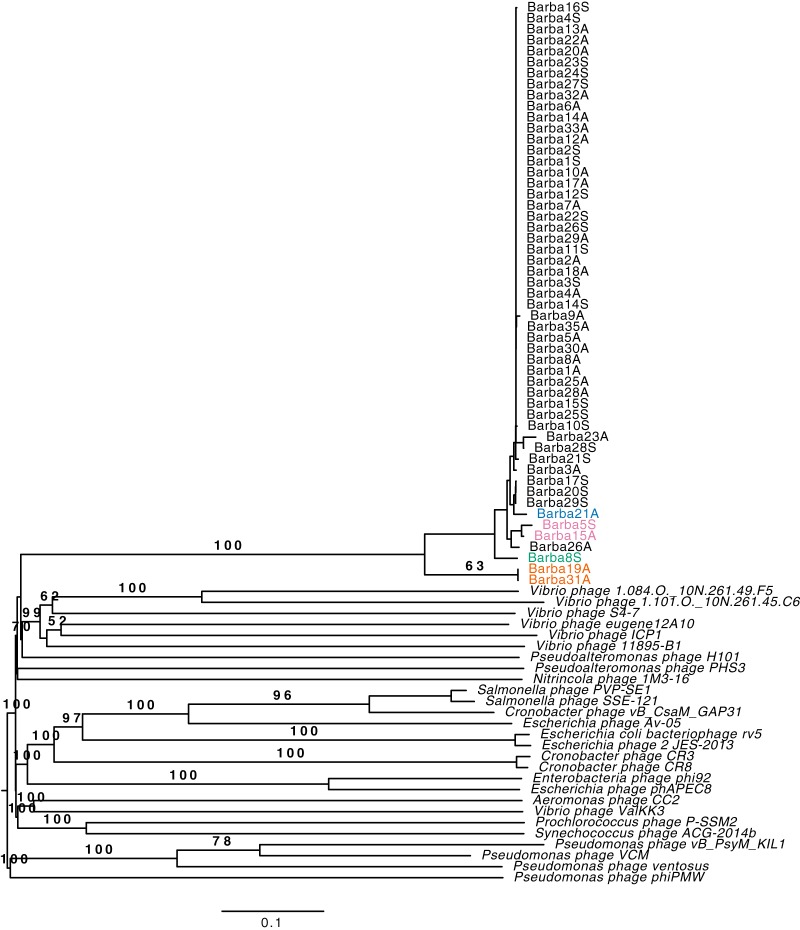
Genome BLAST distance phylogeny (GBDP) tree created with FastME (SPR branch swapping), as a part of VICTOR (42). The numbers above branches are GBDP pseudobootstrap support values from 100 replications (values of >50 are reported), and the branch lengths are scaled in terms of the D0 distance formula (settings: word length, 11; E value filter, 1.0; algorithm, greedy-with-trimming). Isolates belonging to *Barbavirus* are colored based on the species to which they belong as described in the legend of Fig. 1.

Fifty-four phage isolates, originating from individual plaques, infecting *Rheinheimera* sp. strain BAL341 were isolated from the long-term sampling station Linnaeus Microbial Observatory (LMO) in the Baltic Sea Proper (38, 39); 31 isolates were obtained in August 2015, and 23 were obtained in September 2015. The proposed names of the isolates, following standards of the International Committee on the Taxonomy of Viruses (ICTV) (40), take the form *Rheinheimera* phage vB_RspM-barba, followed by numbers indicating the order of isolation and A or S, representing the time of sampling (August or September), e.g., *Rheinheimera* phage vB_RspM-barba18A (see Table S1 in the supplemental material). All 54 phage isolates were whole-genome sequenced, and the genomes were assembled with a coverage ranging from 193× to 4,789× (Table S1). Identical *k*-mers at both ends of the genomes indicated that they were circular. The phage genome sizes varied between 80 and 84 kb, with between 134 and 145 predicted genes in each genome. All-versus-all genome comparison showed that the barba phages shared more than 70% average nucleotide identity (ANI) (Fig. 1; Table S2) across their entire genomes, indicating that they belong to the same genus (>50% ANI) (41). Phylogenetic analysis using VICTOR (42) showed that the isolated phages formed a separate clade that was distinct from previously sequenced and characterized phages (Fig. 2; Table S3). Whereas the low similarity to the reference genomes prevents us from drawing firm conclusions between the novel phages and the reference genomes, the short branches within the clade containing the novel phages let us propose that these phages should be assigned to a novel genus, for which the name *Barbavirus* (Baltic Sea *Rheinheimera* strain BAL341) is suggested. Within this novel genus, 48 of the phage genomes shared more than 95% ANI and therefore belong to one species (Fig. 1; Table S2). Using this species cutoff, four additional species were distinguished, with two species represented by two isolates and two species represented by only a single isolate each (Fig. 1; Table S2). This agreed with the VICTOR analysis for which five species within this one genus were identified (Fig. 2; Table S3). The five species were named based on their suggested type phages: (i) *Rheinheimera* virus Barba18A, (ii) *Rheinheimera* virus Barba21A, (iii) *Rheinheimera* virus Barba5S, (iv) *Rheinheimera* virus Barba8S, and (v) *Rheinheimera* virus Barba19A (Fig. 1). Hereafter, species are indicated with full species name, i.e., *Rheinheimera* virus Barba18A, while isolates are indicated with their unique identifier, e.g., barba18A; also, we use the term barba phages to denote all isolates collectively while *Barbavirus* indicates the species within the genus.

### Morphology and replication characteristics.

**FIG 3:**
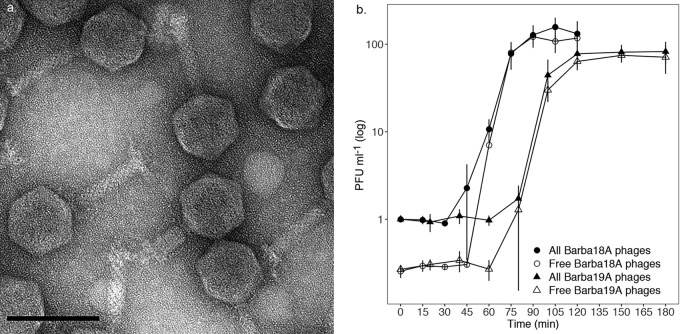
(a) TEM of barba18A shows a myovirus morphology with a capsid that was measured to 72.1 nm (standard deviation, ±2.7 nm), tail length of 88.7 nm (±2.2 nm), and width of 18.8 nm (±1.5 nm). Scale bar, 100 nm. (b) One-step growth curve of barba18A- and barba19A-infected *Rheinheimera* sp. strain BAL341. All phages, phages within and attached to cells as well as unattached phages; free phages, only unattached phages. The values are normalized based on the concentration of all phages at time zero for each replicate. Error bars indicate standard deviations (*n* = 3) but are not shown for barba18A at 60 min for free phages as the deviation was too large (±7.18 PFU/ml).

All phages displayed similar plaque morphologies, with round, clear plaques up to 4 mm in diameter that appeared within 24 to 48 h. Transmission electron microscopy (TEM) of barba18A, the type phage of the genus, showed a myovirus morphology (Fig. 3a), with a capsid diameter of 72.1 nm (standard deviation, ±2.7 nm), a tail length of 88.7 (±2.2) nm, and a tail width of 18.8 (±1.5) nm (*n* = 50). When grown in Zobell medium and replicating on *Rheinheimera* sp. strain BAL341, barba18A had a burst size of 162 (±20) phages and a latent period of 50 (±8.7) min (*n* = 3) (Fig. 3b). barba19A, the type phage of the most divergent species (Fig. 1 and 2), had a significantly smaller burst size of 101 phages (±17 phages; *n* = 3; Welch two-sample *t* test, df = 3.88 and *P* = 0.017) and longer latent period of 80 min (±0 min; *n* = 3; Welch two-sample *t* test, df = 2 and *P* = 0.027) (Fig. 3b).

We found three bacterial strains in our in-house bacterial collection (namely *Rheinheimera* sp. strain BAL331, *Rheinheimera* sp. strain BAL335, and *Rheinheimera* sp. strain BAL336) that had 97 to 99% 16S rRNA gene sequence identity to other cultured *Rheinheimera* bacterial strains in NCBI (Table S4). Compared to the model bacterium *Rheinheimera* sp. strain BAL341, they had 95 to 98% sequence identity on the sequenced parts of the 16S rRNA gene (Table S4). Neither barba18A nor barba19A could infect any of these other *Rheinheimera* strains.

### Gene functionality.

The core genome shared among the 54 novel phage genomes consisted of 97 genes with at least 70% amino acid identity, while the flexible genome consisted of 99 additional genes, most of which were found in *Rheinheimera* virus Barba19A. Thus, the pangenome of these phages was made up of a total of 196 genes (Table S5). Of the genes in the pangenome, 81 genes had significant (E value of <0.001) sequence identity to proteins in the NCBI nonredundant (nr) database, where 81% of the matches were to either gammaproteobacteria or phages infecting bacteria within this class (Table S5). Combined with matches to the pfam database (adding function to 16 additional genes), 43 genes could be assigned to a predicted function (Table 1; Table S5). No tRNAs were detected in the genomes.

**TABLE 1:** Predicted genes with their functions and the functional group to which they belong

Gene name	Length of protein (aa)^*a*^	Functional group^*b*^	Function
Barba18A_gp001	334	Nucleotide metabolism and recycling	Ribonucleotide reductase
Barba18A_gp002	951	Nucleotide metabolism and recycling	Ribonucleotide reductase
Barba18A_gp004	223	Nucleotide metabolism and recycling	Thymidylate synthase
Barba18A_gp005	85	Nucleotide metabolism and recycling	Glutaredoxin
Barba18A_gp006	119	Host signaling	*mazG*
Barba18A_gp008	404	DNA processing, replication, and recombination	Metallo-dependent phosphatase
Barba18A_gp012	202	DNA processing, replication, and recombination	Homing endonuclease HNH
Barba18A_gp013	350	DNA processing, replication, and recombination	Putative exodeoxyribonuclease
Barba18A_gp022	730	DNA processing, replication, and recombination	DNA polymerase
Barba18A_gp023	631	DNA processing, replication, and recombination	DNA primase
Barba18A_gp030	164	Host signaling	macro domain protein
Barba18A_gp071	271	DNA processing, replication, and recombination	DNA adenine methylase
Barba18A_gp072	228	Peptidase	CLP_protease
Barba18A_gp073	262	Phosphate metabolism	*phoH*
Barba18A_gp082	102	Structural and packaging	DNA-binding motif containing protein
Barba18A_gp090	205	Structural and packaging	Putative DNA binding protein
Barba18A_gp095	455	Structural and packaging	Terminase large subunit
Barba18A_gp096	491	Structural and packaging	Putative portal protein
Barba18A_gp097	469	Peptidase	Peptidase
Barba18A_gp099	352	Structural and packaging	Major capsid protein
Barba18A_gp104	359	Structural and packaging	Tail sheath protein
Barba18A_gp105	154	Structural and packaging	Putative structural protein
Barba18A_gp108	213	Structural and packaging	Putative DNA binding protein
Barba18A_gp109	566	Structural and packaging	Tail length tape measure protein
Barba18A_gp113	222	Structural and packaging	Putative baseplate assembly protein
Barba18A_gp116	504	Structural and packaging	Baseplate
Barba18A_gp118	220	Structural and packaging	Tail fiber
Barba18A_gp119	414	Structural and packaging	Tail fiber
Barba18A_gp123	121	Peptidase	Peptidase M15
Barba19A_gp006	82	Nucleotide metabolism and recycling	Glutaredoxin
Barba19A_gp007	190	Host signaling	*mazG*
Barba19A_gp009	209	DNA processing, replication, and recombination	Putative DNA binding protein
Barba19A_gp010	338	DNA processing, replication, and recombination	Metallo-dependent phosphatase
Barba19A_gp033	496	Pyridine nucleotide salvage	Nicotinate phosphoribosyltransferase
Barba19A_gp034	268	Pyridine nucleotide salvage	Ribose-phosphate pyrophosphokinase
Barba19A_gp035	167	Host signaling	Macrodomain protein
Barba19A_gp061	322	DNA processing, replication, and recombination	Nucleotidyltransferase
Barba19A_gp063	419	DNA processing, replication, and recombination	tRNA nucleotidyltransferase
Barba19A_gp098	219	Structural and packaging	Putative DNA binding protein
Barba19A_gp103	427	Structural and packaging	Terminase large subunit
Barba23A_gp115	374	DNA processing, replication and recombination	ISAs1 family transposase
Barba5S_gp006	303	Nucleotide metabolism and recycling	Thymidylate synthase
Barba5S_gp080	254	Phosphate metabolism	*phoH*

a aa, amino acids.

b Genes in the “Structural and packaging” group belong to the structural module, and the other functional groups belong to the host interaction module. For details, see Table S5 in the supplemental material.

**FIG 4:**
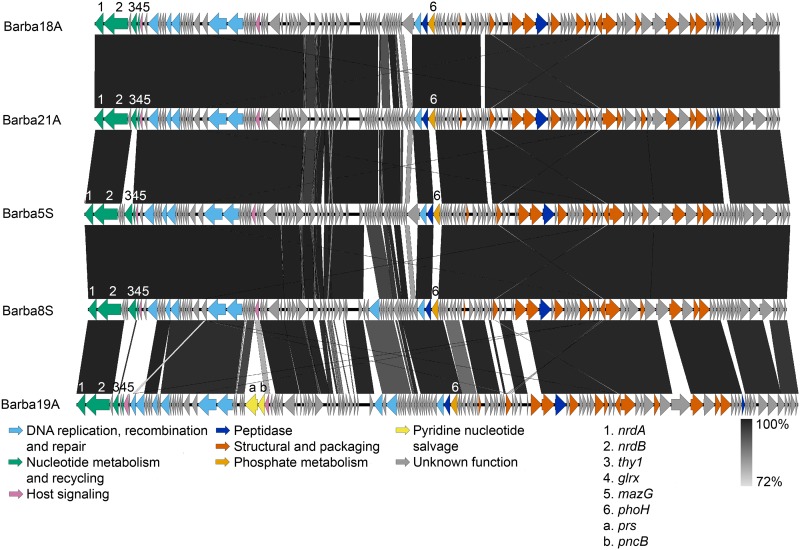
Comparison of genomes from the type phages of the five species within the novel genus *Barbavirus*. Arrows indicate predicted genes, colored by functionality, and the shade of gray between the genomes indicates percent nucleotide identity (blastn), according to the legend on the figure. The metabolic genes and *mazG* discussed in the text are indicated with numbers (1 to 6) or letters (a and b) if they exist in all species or only one, respectively. *nrdA*, ribonucleotide reductase alpha subunit; *nrdB*, ribonucleotide reductase beta subunit, *thy1*, thymidylate synthase; *glrx*, glutaredoxin; *mazG*, nucleoside triphosphate pyrophosphohydrolase; *phoH*, protein PhoH; *prs*, ribose-phosphate pyrophosphokinase; *pncB*, nicotinate phosphoribosyltransferase. The figure was created with EasyFig (106).

The genomes of the five species were similarly arranged and consisted of two larger modules, one for structural genes and one for host interaction genes (Fig. 4). Within the structural module, genes involved in the structure of the phage (e.g., capsid proteins and tail elements), packaging of DNA (e.g., large terminase subunit), and peptidases were detected (Table 1; Table S5). The host interaction module consisted of genes involved in, for example, DNA processing, replication, and recombination (e.g., DNA polymerase and primase), host signaling (nucleoside triphosphate pyrophosphohydrolase [*mazG*]), and various metabolic processes (e.g., ribonucleotide reductase [RNR], thymidylate synthase [*thy1*], and protein PhoH [*phoH*]) (Table 1; Table S5). Of the metabolic genes, two were part of the core genome, the RNR genes *nrdA* and *nrdB*. These genes had 99.9 to 100% amino acid sequence identity in all barba phage isolates. The other metabolic genes found in the barba phages, glutaredoxin (*glrx*) and *thy1*, involved in nucleotide metabolism, and *phoH*, potentially involved in phosphate metabolism, were part of the flexible genome. For these genes, two different variants were detected among the barba phages (Table 2), with the common version shared among all phages except the phages within one particular species, either *Rheinheimera* virus Barba19A or *Rheinheimera* virus Barba5S (Table 2). The genes annotated as *glrx* in barba18A (the common one) and in barba19A (the rare one) shared 43% amino acid identity across 92% of the genes (Table 2). Similarly, the common and rare (in barba5S) versions of the genes annotated as *phoH* shared 31% amino acid similarity across 94% of the genes. However, the two versions of the thymidylate synthase gene did not show significant sequence identity (by blastp, no E value of <0.001) between the two different phage variants (Table 2). In addition, the *Rheinheimera* virus Barba19A isolates contained two metabolic genes that the other isolates lacked, a ribose-phosphate pyrophosphokinase (*prs*) and a nicotinate phosphoribosyltransferase (*pncB*) that are involved in pyridine nucleotide salvage. These phage genes, potentially involved in different metabolic processes, either showed low sequence similarity matches (<40% amino acid identity at an E value of <0.001) or did not provide any significant matches (no E value of <0.001) to genes with similar functions within the host genome (Table 2).

**TABLE 2:** Alignment of genes with metabolic functions within the pangenome of the barba phages^*a*^

Function	Common gene		Rare gene					Best matching host gene against common gene^*d*^				Best matching host gene against rare gene^*d*^			
	No. of isolates	Gene name	No. of isolates	Gene name	E value	% identity	% coverage	Gene name	E value	% identity	% coverage	Gene name	E value	% identity	% coverage
Thymidylate synthase	52	Barba18A_gp004	2	Barba5S_gp006				fig|67575.7.peg.3030				fig|67575.7.peg.2541			
Glutaredoxin	52	Barba18A_gp005	2	Barba19A_gp006	5.00E−20	43	92	fig|67575.7.peg.2555	6.00E−04	27	88	fig|67575.7.peg.1895	4.00E−05	25	96
Protein PhoH	52	Barba18A_gp073	2	Barba5S_gp080	1.00E−42	31	94	fig|67575.7.peg.2145	5.00E−26	28	83	fig|67575.7.peg.2145	4.00E−31	35	80
Ribonucleotide reductase *nrdB*	54	Barba18A_gp001	—^*b*^					fig|67575.7.peg.2913	4.00E−06	22	54				
Ribonucleotide reductase *nrdA*	54	Barba18A_gp002	—^*b*^					fig|67575.7.peg.2912	8.00E−10	23	29				
Nicotinate phosphoribosyl-transferase	2	Barba19A_gp033	—^*c*^					fig|67575.7.peg.3024							
Ribose-phosphate pyrophosphokinase	2	Barba19A_gp034	—^*c*^					fig|67575.7.peg.1160	2.00E−08	27	79				

a Isolate barba18A was used as the subject in phage-phage alignments where the rare gene variant was compared to the common gene, while the host was used as the query for the phage-host alignment. Only significant alignments (E value of <0.001) are included. Alignment was performed using blastp.

b All phages have the same gene.

c No other phages had this gene.

d Determined by blastp.

### Temporal variations.

The barba phages were isolated at two different time points, 19 August 2015 and 16 September 2015. The majority of the isolates belonged to *Rheinheimera* virus Barba18A and were isolated from both time points (Fig. 1). In addition, the two *Rheinheimera* virus Barba5S phages were isolated in both August and September (Fig. 1). Phages belonging to the other species were isolated in either August (*Rheinheimera* virus Barba21A and *Rheinheimera* virus Barba19A, one and two isolates, respectively) or September (*Rheinheimera* virus Barba8S, one isolate).

**FIG 5:**
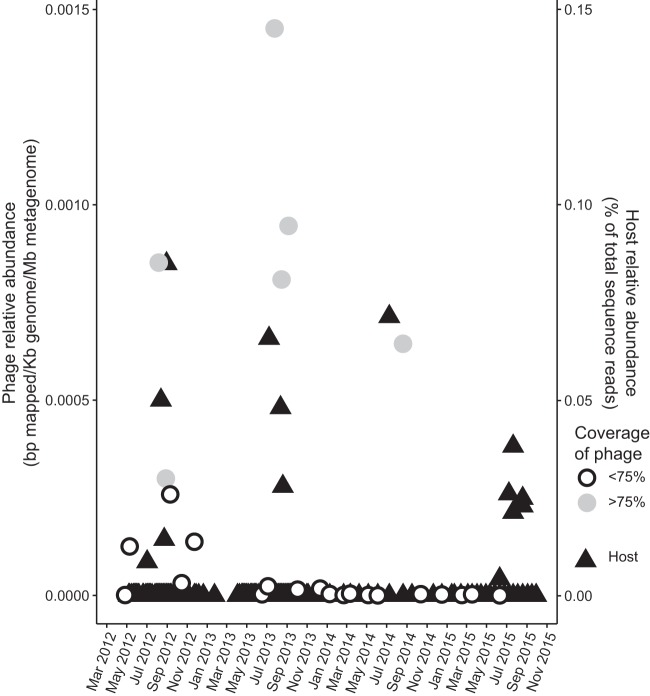
Temporal variation of barba19A and relative abundances of 16S rRNA gene amplicon sequences similar to those of the bacterial host *Rheinheimera* sp. strain BAL341. Circles indicate phage coverage within the sample.

The seasonal dynamics of *Barbavirus* were investigated by competitive recruitment of viral metagenomic reads from the LMO station against the genomes of the five representative phages of each species (barba18A, barba21A, barba5S, barba8S, and barba19A) (Table S6). In order for a phage to be counted as present in a metagenomic data set, at least 75% of a phage genome should be covered by viral metagenomic reads with 90% nucleotide identity (5, 43). According to this definition and using the competitive recruitment, barba19A was detected at one time point while the other four species did not have enough coverage at any time point (Table S6). Due to the high sequence similarity among the barba phages, competitive recruitment was unable to distinguish the true source of reads that belong to the identical parts of the genomes and thus was unable to convey the prevalence of the phages *in situ*. Therefore, barba19A was selected to represent the barba phages and was used for individual recruitment analysis, which resulted in detection of barba19A (<75% coverage) in August and September samples and in one July sample (Fig. 5). The seasonal dynamics of host abundance in the Baltic Sea was determined by quantification of a 16S rRNA gene amplicon sequence variant (ASV) with 100% sequence identity to *Rheinheimera* sp. strain BAL341. These results showed increasing relative host abundances in June to August between 2012 and 2015, which largely mirrored or slightly preceded the phage dynamics (Fig. 5).

## DISCUSSION

In this study, we describe a previously unknown phage genus, all of whose members infected the genome-sequenced bacterial isolate *Rheinheimera* sp. strain BAL341. Genetically, the phage genes showed highest similarity to different gammaproteobacteria or phages infecting gammaproteobacteria. Several of the best viral matches, in particular among genes within the structural module, were to myoviruses (see Table S5 in the supplemental material), which, together with the morphological characterization, placed this genus within the family of *Myoviridae* (Fig. 3a). The head and tail width were of average size compared to the size of other myoviruses, whereas the tail length was relatively short (44, 45). Replication studies of the type phage of the genus, barba18A, and the type phage of the most divergent species, barba19A, showed a burst size (162 and 101 phages, respectively) and latent period (50 and 80 min, respectively) within ranges that are commonly seen among marine phages (17, 25, 46–49). These myoviruses do not fall into any previously known genera and do not share enough sequence similarity to the most similar phages to be able to determine which are their closest evolutionary relatives (Fig. 2). Therefore, we propose that this novel phage genus should be named *Barbavirus*.

All 54 barba phages belonged to the same genus, and thus their diversity appeared limited compared to the genetic diversity seen among phages infecting, e.g., human-associated Escherichia coli and Pseudomonas aeruginosa as well as the Baltic Sea bacterium Cellulophaga baltica, where phages belonging to multiple genera and even different families have been isolated (50). Potentially, the limited diversity within the barba phages could be due to the use of a single host strain for isolation compared to, e.g., 20 host strains used for the C. baltica system (51). This limited diversity is also known from other phage-host systems where single host strains have been used for isolation of phages, e.g., among phages infecting *Roseobacter* strain SIO67 (52) and Bacillus thuringiensis (53). However, it should be taken into consideration that a smaller number of phages were isolated for these systems than of barba phages for the *Rheinheimera* model system. On the other hand, multiple phage genera and even phages of different families have been isolated for other model systems using an individual host strain (47, 54–56) also when few phages were isolated (47). Thus, the use of one individual host strain for isolation cannot be the sole explanation for the limited diversity noted among the barba phages. Instead, this isolation of only highly similar phages might suggest that the barba phages are the dominant viral predators for *Rheinheimera* sp. strain BAL341, as observed during late summer 2015. Further, the high nucleotide similarity among the barba phages might be a result of ecological constraints, such as the reliance on host genes, imposed on the phages while infecting *Rheinheimera* sp. strain BAL341.

The majority (78%) of the pangenome of the barba phages could not be given a predicted function, but the genes for which a functional annotation could be provided showed a genome organization within the barba phages that is typical among phages (22). One module contained genes involved in host interactions, and one module contained genes involved in phage structure and packaging. The host interaction module contained a few genes involved in DNA replication, for example, DNA primase, DNA polymerase, and DNA binding protein. Such genes are commonly found in phages (52, 55) and are involved in the replication of the phage DNA. However, several functions needed for DNA replication were not detected through amino acid similarity searches; potentially, the phage could use host genes for those functions (57, 58), or these functions are among the large number of genes without a predicted function.

The barba phage genomes contained several genes potentially involved in metabolic processes, which are of interest since they can increase the amount of phage progeny (59). Genes involved in nucleotide metabolism, e.g., RNR and thymidylate synthase, are commonly detected within phage genomes and viral metagenomes (60–62). This includes aquatic myoviruses, for example, T4-like cyanophages (61, 63) and *Vibrio* phage KVP40 (13). All barba phages have a complete class I RNR, consisting of the *nrdA* and *nrdB* genes, a glutaredoxin gene, and thymidylate synthase, which are all clustered close together in the genome (Fig. 4). The proteins encoded by the RNR genes *nrdA* and *nrdB* form an enzyme which reduces ribonucleotides to deoxyribonucleotides (64). The class I enzyme is thereafter reduced to its active form by either glutaredoxin or thioredoxin (64), which for the barba phages would suggest the use of the phage-encoded glutaredoxin. Another key part of nucleotide metabolism is thymidylate synthase, the enzyme that converts deoxyuridine 5′-monophosphate to deoxythymidine 5′-monophosphate (65) and is thus an important function that allows phages to be self-sustaining on the nucleotide dTTP. The barba phage gene is most similar to *thy1* (*thyX*), which has the greatest efficiency in the presence of FAD (65). Thymidylate synthase is commonly found among other marine phages, in particular, in relatively large (>70 kb) T4-like cyanomyophages (14), N4-like *Roseobacter* phages (66), and large sipho- and podophages infecting C. baltica (50). These genes, thymidylate synthase, RNRs, and glutaredoxin, all potentially involved in different parts of the nucleotide metabolism, would provide the barba phages with the potential to utilize their own genes to acquire the metabolites they need for DNA replication if the host nucleotide metabolism is not sufficient or is downregulated during phage infection.

The *Rheinheimera* virus Barba19A isolates also contained two additional genes potentially involved in pyridine nucleotide salvage, encoding a ribose-phosphate pyrophosphokinase and a nicotinate phosphoribosyltransferase (NAPRTase) (67). Ribose-phosphate pyrophosphokinase is an enzyme that transforms ribose 5-phosphate and ATP to phosphoribosyl pyrophosphate (PRPP) and AMP (68). PRPP is a substrate in several enzymatic processes in bacteria (69) and is a precursor of purine and pyrimidine nucleotides, as well as the pyridine nucleotide NAD^+^ (70). Purine and pyrimidine are used by RNR to produce the nucleotides that are needed during the replication of the phage, whereas within the pyridine nucleotide salvage pathway, PRPP and nicotinic acid are converted by NAPRTase to nicotinate mononucleotide and pyrophosphate, most efficiently by hydrolysis of ATP (67). Nicotinate mononucleotide can then be converted to NAD^+^, which is involved in amino acid catabolism and DNA ligase reactions and is a key coenzyme in redox reactions where its reduced form (NADH) transfers electrons to the electron transport chain (71). However, the function within phages has not been clarified. A complete pyridine nucleotide salvage pathway, with *nadV* and *natV*, is functional in *Vibrio* phage KVP40, where *nadV* has functional similarity to NAPRTase (72). The *nadV* gene has also been found in other phages, including another *Vibrio* phage (73) and a marine *Pseudoalteromonas* phage (74). The reaction of *nadV* is followed by the conversion of nicotinamide mononucleotide to NAD^+^ by *natV* in the presence of ATP (72), for which a homologue is missing in *Rheinheimera* virus Barba19A. While the particular functionalities of the ribose-phosphate pyrophosphokinase and the NAPRTase in the *Rheinheimera* virus Barba19A phages have not been verified, these extra metabolic gene products could be hypothesized to provide this species a fitness advantage over the other barba species by increasing the efficiency of the RNRs through redox reactions. However, this is not evident for replication on *Rheinheimera* sp. strain BAL341, where barba19A had a significantly smaller burst size and longer latent period than barba18A. It should be taken into consideration that during the replication experiment, the nutrient supply was high and did not correspond to *in situ* conditions, rendering it hard to detect the increased metabolic potential of barba19A that might be evident as a fitness advantage in the Baltic Sea.

All barba phage genomes contained a *mazG* homologue, even though the *Rheinheimera* virus Barba19A gene showed no sequence similarity to the gene in the other phages (Tables 1 and 2). MazG is predicted to halt self-programmed cell death (75) and has been hypothesized to be used by the phage to keep the host alive during phage propagation (76). A functional MazG acts by decreasing the cellular pool of guanosine 3′,5′-bispyrophosphate (ppGpp), which changes the metabolism of the cell as a stress response, that in turn will halt the toxic effects of MazF by resynthesizing MazE (75). Bioinformatic analysis has suggested that MazG is overrepresented among marine phages (22), such as the marine *Pseudoalteromonas* phage H105/1 (22), Roseophage SIO1 (52), and T4-like cyanophages (77). Therefore, MazG has been suggested to have an important role in marine phage systems (22). Also, *phoH* was detected in all barba phages even though the *phoH* gene in *Rheinheimera* virus Barba5S differed from the version found in the other barba phages. In E. coli, *phoH* is part of the Pho regulon, where it is expressed during phosphate limitation, potentially to transport or use phosphate (78). However, a bioinformatic approach to investigate the function of PhoH suggests that the protein could also be involved in phospholipid metabolism and RNA modification or fatty acid beta-oxidation (79). *phoH* has been shown to be upregulated during late infection of the cyanobacterium *Prochlorococcus* strain MED4 by cyanophage P-SSP7 (80). This upregulation is speculated to be a reaction to stress in the host due to phage infection (80) or a consequence of P limitation caused by phage production (27), where an increased uptake of phosphorus would increase phage replication success. Even though the exact mechanism of the *phoH* gene is not fully determined, the gene appears to be more important for marine phages than for phages from other environments; the *phoH* gene is present in 40% of sequenced marine phages in contrast to 4% of phages from other environments (81). The presence of both *mazG* and *phoH* in the phage genome might be indicative of adaptations to counter the host response to stressors caused by the phage infection and might increase the quantity of newly created phages.

Phage metabolic genes can be used to increase replication success and might originate from the host’s genome. However, with respect to the barba phages, their metabolic genes were distantly related to genes of similar function occurring in the host *Rheinheimera* sp. strain BAL341 (Table 2). For example, the RNR genes within the barba phages shared low sequence similarity to the host’s RNR genes (23% for *nrdA* and 22% for *nrdB*) (Table 2). In general, phages have the same RNRs as their host, but the opposite has also been noted in several instances (60, 82) and has been suggested to be a sign of either horizontal gene transfer within phages with extended host range or of a shifted host range in phages over longer time periods (83), which potentially could be the case for these genes in the barba phages.

The phages isolated in August and September were highly similar at both the nucleotide and amino acid levels, and there were no obvious indications that this phage population had changed genetically between August and September. Within the temporal viral metagenomes from 25 different samplings between 2012 and 2015, the barba phages, represented by barba19A, were detected from July until September, when their host was either abundant or had just declined (Fig. 5). The relative abundance of barba19A within this viral metagenomic data set was low compared to that of investigated uncultured phages within marine metagenomes (5), but the recurring presence of barba19A during the entire investigated time frame indicates that the barba phages form a stable part of the Baltic Sea phage community in late summer (Fig. 5).

While barba19A was the species that was covered by the largest number of viral metagenomic reads during a competitive recruitment (Table S6) and therefore represented the abundance of the barba phages in the temporal data set (Fig. 5), *Rheinheimera* virus Barba18A had the most associated isolates (Fig. 1). This could be due to a shift in the phage community whereby *Rheinheimera* virus Barba19A dominated the barba phage population during the earlier years, as seen by coverage of viral metagenome reads, while *Rheinheimera* virus Barba18A was the dominant species at the time points of isolation and, hence, had a large number of associated isolates. On the other hand, *Rheinheimera* sp. strain BAL341 may not be the optimal or preferred host for the wild-type *Rheinheimera* virus Barba19A phages, and therefore not as many plaques were retrieved as for *Rheinheimera* virus Barba18A. This could explain the reduced replication success for barba19A when it replicates on BAL341 compared to that of barba18A (Fig. 3b). Similar behavior is known from aquatic *Cellulophaga* phages replicating on nonoptimal hosts (16, 17). Efficiency of infection can be another indication of host suitability, but based on the one-step growth curves (Fig. 3b, difference between total and free phages before the burst), efficiency levels were similar for barba18A and barba19A. Yet efficiency of infection is highly linked to the host bacterium that was latest infected (16), and thus a high efficiency of infection of our isolated barba19A, which has been passed through *Rheinheimera* sp. strain BAL341 multiple times, might not be representative of the efficiency of infection of the wild-type barba19A. However, no alternative host could be detected for barba18A and barba19A within our bacterial culture collection, but it should be noted that the range of suitable alternative hosts was limited. Still, *Rheinheimera* virus Barba19A is the species that could be detected in the Baltic Sea during several consecutive years (Fig. 5; Table S6), and potentially their additional metabolic genes provide them with the fitness advantage to make them the dominant member of the *Barbavirus* population.

Whether or not barba18A or barba19A was the dominant barba phage species during 2015, these replication differences and the temporal dynamics of phages and their host seen over multiple years are indicative of a within-genus phage variation, where different phage species will have varied impacts on their host strains. The pattern of increased phage abundance that coincides with the increase of host abundance, measured as relative abundance of 16S rRNA gene amplicon reads (Fig. 5), is representative of the Lotka-Volterra-like phage-host oscillations suggested in the “kill-the-winner” hypothesis (84). The yearly, reoccurring increases of *Rheinheimera* sp. strain BAL341 and its phages after the summer cyanobacteria bloom (38) point toward the ecological importance of this phage-host system connected to bloom degradation or the exudates excreted during bloom declines. Given its ecological relevance and the high number of metabolic genes, the *Rheinheimera* phage-host system represents an important novel model system for hypothesis-driven research, such as describing different genes of unknown function, how the expression of metabolic genes during infection under various growth conditions influences phage progeny, and how the phage replication success is affected by different environmental factors, such as temperature or nutrient concentrations. These are important aspects to consider for an increased understanding of phage-host interactions and the ecological implications of these microbial players in aquatic environments.

## MATERIALS AND METHODS

### Bacterial isolation.

Water for bacterial isolation was collected at a 2-m depth at LMO (56°55.8540′N, 17°3.6420′E), situated 10 km east off the coast of Öland, Sweden, with a Ruttner sampler on 12 July 2012. Bacterial colonies were grown on Zobell agar plates (1 g of yeast extract [Becton, Dickinson and Company (BD)], 5 g of Bacto peptone [BD], and 15 g of Bacto agar [BD] in 800 ml of filtered Baltic Sea water and 200 ml of Milli-Q water) at room temperature for 3 to 4 days, and each isolate was clean streaked three times. Bacterial DNA for sequencing of the 16S rRNA gene (BAL331, BAL335, BAL336, and BAL341) and for whole-genome sequencing (BAL341) was isolated using an EZNA tissue DNA kit (Omega Bio-tek) according to the manufacturer’s instructions. Briefly, cells were lysed, protease treated, and bound to a HiBind DNA Mini column. While bound to the column, the DNA was washed with DNA wash buffer diluted with 100% ethanol and then eluted in 10 mM Tris. The 16S rRNA gene was PCR amplified with the primers 27F and 1492R and sequenced using Sanger dideoxy sequencing at Macrogen Europe, Amsterdam, Netherlands. The whole-genome DNA was verified with regard to quality and quantity with Nanodrop (Thermo Scientific) and Qubit (high-sensitivity DNA kit; Invitrogen, Life Technologies), respectively, and sequenced with an Illumina HiSeq system at the Science for Life Laboratory, the National Genomics Infrastructure (SciLife/NGI) (see the paragraph “Sequencing of whole genomes” below).

### Viral isolation.

Water for viral isolation was collected on 19 August 2015 and 16 September 2015 at LMO. At each time point, 10 liters of water was prefiltered through 0.22-μm-pore-size Sterivex filters (Millipore), and the water was concentrated using a 30-kDa tangential flow filter (Millipore). The resulting 100 to 150 ml was further concentrated through 50-kDa Amicon Ultra centrifugal filters (Millipore) to 13 to 30 ml.

In order to isolate phages, plaque assays (85) with the two viral concentrates were conducted using *Rheinheimera* sp. strain BAL341 as the host. Here, bacteria grown overnight in liquid Zobell medium (1 g of yeast extract [BD] and 5 g of Bacto peptone [BD] in 800 ml of filtered Baltic Sea water and 200 ml of Milli-Q water) were mixed with the viral concentrates and molten (32°C) top agar (marine sodium magnesium [MSM] buffer consisting of 450 mM NaCl [Sigma], 50 mM MgSO_4_·7H_2_O [Fisher], and 50 mM Trizma base [Sigma], pH 8, with 0.5% low-melting-point agarose [Thermo Fisher Scientific]). The mixture of bacteria, viral concentrate, and molten agar was spread evenly onto Zobell agar plates. The plates were incubated at room temperature (RT) for 2 days, and plaque formation was monitored on a daily basis. To obtain pure phage isolates, individual plaques (54 in total) were picked with a sterile 100-μl pipette tip, dispersed in MSM buffer, and replated three times as described above, but now with phages suspended in MSM buffer and not viral concentrates. Pure isolates were harvested by adding 5 ml of MSM buffer to fully lysed plates, the top agar layer was shredded with an inoculation loop, and the plates were incubated on a shaking table (40 rpm) for at least 30 min. The MSM buffer with suspended phages was collected into a Falcon tube, centrifuged for 10 min at 10,000 × *g*, filtered through a 0.2-μm-pore-size syringe filter (BD), and stored at 4°C.

### TEM.

Phage particles were purified by cesium chloride (CsCl_2_) density gradient centrifugation (described by Carlson and Miller [86]), with modifications as described below, and analyzed by transmission electron microscopy (TEM). Briefly, a high titer of phage lysate (∼10^11^ ml^−1^) was applied to a CsCl_2_ step gradient (densities of 1.1, 0.9, 0.7, and 0.5 g ml^−1^) and centrifuged in a Spinco SW39 rotor for 3 h at 24,000 rpm (280,000 × *g*), 4°C. The band with the highest opalescence was collected with a syringe and dialyzed three times with SM buffer (100 mM NaCl, 8 mM MgSO_4_, 50 mM Tris-HCl, pH 7.5) in dialysis tubes (0.020 mm; Viskase, USA) for 24 h at 4°C. The suspension was applied to carbon-coated nitrocellulose grids (Agar Scientific Elektron Technology, United Kingdom), stained with two successive drops of 2% uranyl acetate (pH 4.5), dried, and examined using a Morgagni 268(D) transmission electron microscope (FEI, USA). In total, 50 phage particles were measured for determination of capsid size and tail length using the Morgagni integrated image acquisition and analysis software.

### Host range.

Host range analysis based on efficiency of plating was performed with two species type phages from this study, barba18A and barba19A, on the in-house *Rheinheimera* bacterial strains, *Rheinheimera* sp. BAL341, BAL331, BAL335, and BAL336, isolated from LMO. Plaque assays as described previously were performed in duplicate for each phage-host pair with phage dilutions of 10^−2^- to 10^−4^-fold, except for *Rheinheimera* sp. strain BAL341, for which the phages were diluted from 10^−2^ to 10^−9^, and plates were monitored daily for 5 days to detect plaque formation.

### One-step growth curve.

To determine the exponential growth phase of *Rheinheimera* sp. strain BAL341, the bacterium was grown in 10 ml of Zobell medium in glass tubes at RT in triplicates, and optical density at 600 nm with a CO8000 cell density meter (WPA, Cambridge, United Kingdom) was measured every hour for 14 h. One-step growth curves, providing phage replication characteristics, were performed as described in Holmfeldt et al. (16), with minor modifications. Briefly, exponentially growing bacteria were infected with barba18A or barba19A at a multiplicity of infection (MOI) of 0.1 for 5 min, after which the solution was diluted 1,000-fold in order to prevent new infections. Free and total numbers of phages were enumerated using a plaque assay (described above) every 15 min for 2 h for barba18A and every 20 min for 2 h and then every 30 min for one additional hour for barba19A. To enumerate free (extracellular) phages, subsamples of the diluted infection were filtered through a 0.2-μm-pore-size syringe filter (BD), and the flowthrough was used for the plaque assay. For total phages, including both extracellular phages and phages attached to or replicating inside bacteria, subsamples were retrieved directly from the incubation and used for plaque assay. Plaque formation was examined within 48 h. Statistical analysis and plotting were done with R (version 3.5.1) (87) through RStudio (version 1.1.383) (88) using the packages plyr (version 1.8.4) (89) and ggplot2 (version 3.1.0) (90).

### Viral DNA extraction.

DNA extraction from the 54 isolated phages was performed with Wizard PCR DNA purification resin and minicolumns (Promega). First, 1 ml of resin was added to 1 ml of the newly harvested, high-titer viral sample, thoroughly mixed, and then pushed through a minicolumn. The column was washed twice using 1 ml of 80% isopropanol, and residues of isopropanol were removed by centrifuging the column for 2 min at 10,000 × *g*. The DNA was then eluted with preheated (80°C) 10 mM Tris (Trizma base [Sigma], pH 8) by first vortexing the column and then centrifuging it at 10,000 × *g* for 30 s. Quality and quantity of the DNA were verified with Nanodrop (Thermo Scientific) and Qubit (high-sensitivity DNA kit; Invitrogen, Life Technologies), respectively.

### Sequencing of whole genomes.

Library preparation of DNA from whole-genome extraction of *Rheinheimera* sp. BAL341 and the 54 viral isolates was done with Nextera XT (Illumina, Inc., San Diego, CA, USA), and sequencing was performed by SciLife/NGI (Solna, Sweden) on a HiSeq 2500 system (Illumina, Inc.), which resulted in paired-end 125-bp reads (average of 1.2 million reads per sample).

### Bioinformatics analysis.

Reads in fastq format were trimmed to remove adapters and poor-quality reads (trimmomatic version 0.30) (settings: -PE –threads 2 -phred33 ILLUMINACLIP:nextera_linkers.txt:2:30:10 LEADING:3 TRAILING:3 SLIDINGWINDOW:4:15 MINLEN:30) (91), and quality was then evaluated with FastQC (92). Reads were assembled with Spades (version 3.6.0) (settings: –careful –t 8 –pe1-1 –pe1-2 -o) (93) and Abyss (version 1.3.5) (settings: -np 16 –k61 –q3 –coverage-hist –s –o) (94), and the assembler providing the longest contig was chosen. The bacterial genome was annotated with the RAST online service (95, 96). The phage genomes were manually inspected, and gene prediction was performed with GeneMark (version 3.26; gene code 11, heuristic parameters as 2010) (97) and manually curated on completed genomes. Predicted genes were then annotated with Diamond (version 0.8.26) (settings: blastp –p 16 –q –k 500 –min-score 30 –sensitive –tmpdir –f 6 -o) (98) against the NCBI nonredundant database (February 2018) and hmmsearch (version 3.1b2) (settings: –tblout –E 1e-3 –cpu 4) (99) against the Pfam database (100) (February 2018, version 31.0), and an alignment was considered significant if the E value was less than 0.001. Also, to identify the phages with highest similarity to the barba phages, a Diamond search with the same parameters as described above was run against a custom viral database (viraldb) consisting of viral genomes from RefSeq (downloaded 14 September 2018). The results from the search against the viraldb were used to define reference genomes for the VICTOR analyses described below. Searches for tRNA were performed with tRNAscan-SE (settings: -qQ –detail -o# -m# -f# -l# -c tRNAscan-SE.conf -s# -B) (101). The core genome and pangenome were calculated with Roary (102), with settings not to split paralogs and to group genes with more than 70% amino acid similarity (-s -i 70). Whole-genome comparisons on the nucleotide level were done with gegenees (version 3.0.0) (settings: fragment size of 500 and step size of 500; blastn) (103) that fragments the genomes and performs all-against-all blastn alignments before calculation of the average nucleotide identity across the entire genomes. The gegenees results were plotted with reshape2 (version 1.4.3) (104) and Tidyverse (version 1.2.1) (105) in R (version 3.5.1) through RStudio (version 1.1.383). Comparison of gene distributions between a select set of genomes was conducted using EasyFig (106).

### Phylogenetic analysis.

To distinguish taxonomic classification of the barba phages from that of other viruses, all 54 barba phage isolates and selected reference genomes, based on alignments to the NCBI nr database and viraldb, were analyzed with VICTOR, a genome-to-genome distance calculator (42). Pairwise comparisons of nucleotide sequences were done by using the genome-BLAST distance phylogeny (GBDP) method (107) under settings for the D0 formula (settings: word length, 11; E value filter, 1.0; algorithm, greedy-with-trimming) recommended for prokaryotic viruses (42). The distances were then used to infer a balanced minimum evolution tree with branch support (subtree pruning and regrafting [SPR] branch swapping, 100 pseudobootstrap replicates) via FastME (108). Also, the genomes were clustered at species, genus, and family levels with the OPTSIL program (109) with recommended thresholds (42) and an *F* value (fraction of links required for cluster fusion) of 0.5 (110).

### Viral metagenome sampling and recruitment to the phage genomes.

At 25 time points between 2012 and 2015 (for dates, see Table S7 in the supplemental material), water was collected at the LMO in the same manner as described above. The water was prefiltered through an 0.22-μm-pore-size filter (Sterivex cartridge filter; Millipore) to remove larger organisms. Viruses were precipitated using the iron chloride method (111), where the flowthrough was treated with FeCl_3_ (final concentration, 1 mg liter^−1^; Sigma) to aggregate the viral particles, which were collected on a 1.0-μm-pore-size filter (polycarbonate; Maine Manufacturing LLC) and stored in falcon tubes at +4°C until DNA extraction. For DNA extraction, the viral particles were resuspended in ascorbate solution (20 ml of 0.5 M EDTA, pH 8, 12.5 ml of 1 M Tris, pH 8, 1.9 g of MgCl_2_, 3.52 g of ascorbic acid, ∼4 ml of NaOH, and Milli-Q water up to 100 ml) within 1 to 18 months after sampling and concentrated using Amicon spin filters (Millipore). The viral concentrates were thereafter treated with DNase I (Invitrogen) according to the manufacturer’s recommendations and inactivated with EDTA (100 mM; Sigma) by incubation at 65°C for 15 min. The viral particles were treated with preheated (37°C for 30 min) proteinase K (20 mg/ml; Fisher) at 37°C for 12 h, and the DNA was then extracted with a Wizard PCR DNA purification kit as described above. The quality and quantity of DNA were assessed with Qubit (Invitrogen) and Nanodrop (Thermo Scientific), respectively, and sequenced at SciLife/NGI (Solna, Sweden). Nine of the viral metagenomes were sequenced during 2014, and the last 16 samples were sequenced during 2016. Libraries for the first set were prepared using Illumina TruSeq DNA, clustered with cbot, and sequenced with a 2-by-101 setup. For the second set, the libraries were prepared using Illumina TruSeq Nano, clustered with cbot, and sequenced with a 2-by-126 setup (Table S7). Both sets were sequenced on an Illumina HiSeq 2500 system (Illumina, Inc., San Diego, CA, USA). Reads from the 25 viral metagenomes were recruited against barba18A, barba21A, barba5S, barba8S, and barba19A competitively and against barba19A exclusively using Bowtie2 (version 2.3.3.1, with default settings) (112). To calculate coverage and depth, samtools (version 1.9; setting -F4) (113) was used to filter out alignments with low quality (<90% identity to the reference), and bedtools (version 2.27.1) (114) was used to perform calculations of coverage and depth. Read depth was normalized based on the length of the genome in kilobases and the size of the viral metagenome in megabases as previously done for other viral metagenome analyses (5).

### Amplicon sequencing and host identification.

Bacterial 16S rRNA gene amplicon sequence data for monitoring host abundance were obtained according to Boström et al. (115), as modified by Bunse et al. (116). Briefly, water samples from LMO (2012 to 2015) were collected and either sequentially filtered through 3.0-μm-pore-size, 48-mm-diameter, polycarbonate filters (Pall Life Sciences) and then through 0.2-μm-pore-size Sterivex cartridge filters (Millipore) or directly through Sterivex cartridge filters (Millipore). DNA was extracted using the phenol-chloroform method (117) from all filters, and the V3-V4 region of the 16S rRNA gene was amplified in PCR with the primer pair 341f-805r (118, 119). Illumina adapters were attached during a second PCR, and the samples were sequenced (300 bp; pair end) on the Illumina MiSeq platform (Illumina, Inc., USA) at SciLife/NGI (Solna, Sweden). Raw reads were subsequently processed with the DADA2 pipeline (120) to produce amplicon sequencing variants (ASVs) (filterAndTrim: –trimLeft = 8,8 –truncLen = 290,210, learnErrors: –randomize=TRUE, –nsamples = 24, mergePairs: –minOverlap = 10, isBimeraDenovo: minParentAbundance = 8, minFoldParentAbundance = 4). Using RStudio (version 1.1.383) (88) and the Tidyverse package (version 1.2.1) (105), the ASV sequence matching *Rheinheimera* sp. strain BAL341 (100% nucleotide identity, by blastn) was extracted from the data set from all three filter fractions (3.0 μm, 0.2 μm, and 0.2 μm after 3.0 filtration) and plotted as relative abundances (proportions) over time together with the phage abundances using ggplot2 (90).

### Data availability.

16S rRNA gene accession numbers for *Rheinheimera* sp. BAL341, BAL331, BAL335, and BAL336 were deposited in GenBank under accession numbers KM586890, KM586876, KM586880, and KM586917, respectively. Sequences for the barba phages are deposited under accession numbers MK719701 to MK719754.

Raw reads for *Rheinheimera* sp. strain BAL341 were deposited in NCBI under BioProject accession number PRJEB29737. Raw sequence reads for the viral metagenome were deposited under BioProject accession number PRJNA474405. Whole-genome and annotation information for *Rheinheimera* sp. strain BAL341 is available in the EMBL/ENA database under accession number CAAJGR010000000.

## Supplementary Material

Supplemental file 1
